# Disease Duration Differentiates Freezing of Gait Response to Subthalamic and Nigral Stimulation for Parkinson's Disease in an Exploratory Multicenter Controlled Trial

**DOI:** 10.1002/mdc3.70813

**Published:** 2026-10-12

**Authors:** Daniel Weiss, Idil Cebi, Marion Schütt, Imma Fischer, Lukas Pavelka, Pierre Kolber, Monika Pötter‐Nerger, Wolfgang Hamel, Andrea A. Kühn, Christian J. Hartmann, Kai Bötzel, Thomas Köglsperger, Michael T. Barbe, Zacharias Kohl, Steffen Paschen, Markus A. Hobert, Sergiu Groppa, Tobias Warnecke, Inga Claus, Rejko Krüger, Alireza Gharabaghi, For the Multicenter STN+SNr Study Group, Christian Stallinger, Frank Hertel, Daniel Kroneberg, Carsten Buhmann, Alessandro Gulberti, Beate Schönewald, Verena Zentsch, Verle Visser‐Vandewalle

**Affiliations:** ^1^ Centre for Neurology, Department for Neurodegenerative Diseases, and Hertie‐Institute for Clinical Brain Research University of Tübingen Tübingen Germany; ^2^ Institute for Clinical Epidemiology and Applied Biometry, University of Tübingen Hamburg Germany; ^3^ Parkinson Research Clinic, Centre Hospitalier de Luxembourg, Luxembourg; and Translational Neuroscience, Luxembourg Centre for Systems Biomedicine (LCSB), University of Luxembourg, Esch‐sur‐Alzette, Luxembourg; and Translational Neuroscience, Luxembourg Centre for Systems Biomedicine (LCSB), University of Luxembourg Esch‐sur‐Alzette Luxembourg; ^4^ Translational Neuroscience, Luxembourg Centre for Systems Biomedicine (LCSB), University of Luxembourg Esch‐sur‐Alzette Luxembourg; ^5^ Department of Neurology, and Neurosurgery University Medical Center Hamburg‐Eppendorf Hamburg Germany; ^6^ Department of Neurology Charité‐Universitätsmedizin Berlin Berlin Germany; ^7^ Institute of Clinical Neuroscience and Medical Psychology, and Department of Neurology, Medical Faculty, Heinrich‐Heine University Düsseldorf Düsseldorf Germany; ^8^ Department of Neurology University Hospital of the LMU Munich Munich Germany; ^9^ Department of Neurology, Faculty of Medicine and University Hospital Cologne, University of Cologne Jülich Germany; ^10^ Cognitive Neuroscience, Institute of Neuroscience and Medicine, Research Centre Jülich Jülich Germany; ^11^ Department of Neurology University of Regensburg Regensburg Germany; ^12^ Department of Neurology, UKSH Campus Kiel Christian‐Albrechts‐University Kiel Germany; ^13^ Department of Neurology, UKSH Campus Luebeck, University of Luebeck Luebeck Germany; ^14^ Imaging and Neurostimulation, Department of Neurology University Medical Center of the Johannes Gutenberg University Mainz Mainz Germany; ^15^ Department of Neurology University of Münster Münster Germany; ^16^ Department of Neurology and Neurorehabilitation Klinikum Osnabrück‐Academic Teaching Hospital of the University of Münster Münster Germany; ^17^ Institute for Neuromodulation and Neurotechnology Tübingen Germany; ^18^ Center for Bionic Intelligence Tübingen Stuttgart, University of Tübingen Tübingen Germany; ^19^ German Center for Mental Health (DZPG) Tübingen Germany; ^20^ Max Planck‐University of Toronto Centre for Neural Science & Technology (MPUTC) Toronto Ontario Canada

**Keywords:** deep brain stimulation, freezing, gait, Parkinson's disease, substantia nigra, subthalamic nucleus

## Abstract

**Background:**

Freezing of gait (FoG) belongs to most pressing unmet symptoms in the treatment of Parkinson's disease.

**Objectives:**

To explore whether combined subthalamic and nigral (STN + SNr) deep brain stimulation improves FoG compared with optimized STN stimulation alone; to identify clinical variables associated with therapeutic response.

**Methods:**

In this exploratory multicenter randomized controlled double‐blind trial, we recruited patients from our existing cohorts and randomized them 1:1 to STN or STN + SNr. After baseline, patients were followed with close interim visits (visits 1–5) for 90 days (visit 6). The Freezing of Gait Assessment Course (FoG‐AC) was the primary endpoint and was evaluated at *P* < 0.2 with exploratory intent. Secondary endpoints assessed responder rates, treatment dynamics, predictors of response, and patient preferences.

**Results:**

At V6, STN (n = 23) and STN + SNr (n = 22) FoG‐AC outcomes did not differ significantly. We identified responders with clinically meaningful FoG improvement in both the STN (n = 7) and STN + SNr (n = 8) groups. In STN + SNr responders, mean disease duration was ~12 years, which was ~4.5 years shorter than in non‐responders, but there were no differences in motor and cognitive functions. Treatment dynamics suggested that FoG responders improved slowly and gradually. The STN + SNr responder group showed numerically stronger FoG improvement compared with STN responders. Additionally, 15 STN compared to 10 STN + SNr patients wished to change treatment after the RCT phase and before unblinding. Both STN and STN + SNr treatment was safe.

**Conclusions:**

Future trials evaluating adjunctive STN + SNr stimulation should stratify patients by disease duration to optimize outcomes.

Freezing of gait (FoG) is among the most pressing unmet therapeutic needs in Parkinson's disease (PD). While in early disease stages FoG is either not present or effectively masked by dopamine replacement therapy, >60% of patients express FoG as the disease progresses.^1^ This is relevant when counseling PD patients for subthalamic nucleus deep brain stimulation (STN‐DBS) therapy,^2^ or if FoG progresses after primarily effective STN‐DBS.^3^, ^4^, ^5^

Effective neuromodulation therapy for FoG has only partly been achieved. The most remarkable benefit was attained in early motor fluctuations, when off‐related FoG is still responsive to dopamine replacement but better controlled for with STN‐DBS.^2^, ^6^ However, STN‐DBS at “higher” frequencies like 130 Hz is not always effective or may even worsen FoG^7^ upon current spreading to the pallido‐thalamic tract, antero‐medial to the STN.^8^ To overcome these limitations, several reprogramming strategies have been explored. “Low frequency” stimulation of the STN, likewise with 60 or 80Hz,^7^, ^9^ was effective in a proportion of patients while others showed worsening of segmental motor symptoms. Further, the effect may habituate weeks to months from reprogramming.^9^ Interleave‐interlink programming of the STN—embedding low‐frequency stimulation interleaved with high‐frequency stimulation on a neighboring contact—showed improved axial motor outcomes based on clinical global impression‐severity ratings (CGI‐S).^10^

We previously introduced the combined stimulation of STN and substantia nigra pars reticulata (SNr),^11^, ^12^ which is located at the interface to the descending locomotor brainstem pathways.^4^, ^12^ As advantage, the primarily effective STN parameters can often be maintained when adding nigral impulses with interleaving stimulation. However, a role of interindividual variation in functional anatomy, and hence electrode location as well as current spreading to adjacent structures like ventromedial STN or SN pars compacta (SNc) are possible in individual patients and may impact on the stimulation effects. While talking from “SNr” in this study, the label should be understood as encompassing these possibilities. In this framework, there was first indication that SNr stimulation modified clinical and kinematic gait‐ and balance‐related parameters.^4^, ^13^ Furthermore, STN + SNr showed variable effect on FoG in single case observations,^14^, ^15^, ^16^ a small case series,^17^ and several controlled studies.^10^, ^12^, ^18^, ^19^, ^20^ STN + SNr was mostly safe while side effects occurred rarely^12^, ^21^ including on swallowing.^22^, ^23^

In this light, we studied the effect of STN + SNr on FoG on our existing cohorts in an exploratory multicenter randomized controlled study. We integrated narrow interim visits to monitor closely the treatment dynamics of STN + SNr. Furthermore, we explored the clinical characteristics of responders.

## Methods

This investigator‐initiated, double‐blind, randomized, controlled multicenter trial (ClinTrials.gov: NCT02588144) intended to enroll 54 patients from 12 expert centers. Importantly, patients were selected from the pre‐existing DBS cohorts of each center. We selected those, in whom the caudal most contacts likely reached the SNr according to pre‐defined standard coordinates. We did not prospectively implant patients with SNr, which was the main reason for restricting the sample size and for powering the primary endpoint to *P* < 0.2 while a confirmatory study would have needed substantially higher sample size. We closed recruitment prematurely once we could not identify more patients with FoG and electrodes reaching the caudal STN + SNr border zone.

We screened 53 patients, 48 patients were enrolled. From these, one was randomized erroneously after initial screening failure and did not receive study‐related treatment. Two patients retracted informed consent during the active study phase. The remaining 45 patients (Table 1) were referred to statistical analyses as modified intention‐to‐treat‐cohort (mITT). The per‐protocol population consisted of 39 patients (20 standard STN, 19 STN + SNr), exclusive of 6 patients with major protocol deviations (Consort Chart).

**TABLE 1:** Baseline characteristics of “STN” and “STN + SNr” groups

	Randomized treatment group (mITT)		
	STN (N = 23) N (%) or mean ± SD	STN + SNr (N = 22) N (%) or mean ± SD	*P*‐value
Gender: female	7 (30.4)	4 (18.2)	0.339
Male	16 (69.6)	18 (81.8)	
Age	68.3 (7.6)	69.7 (6.8)	0.512
Disease duration (years)	15.9 (6.4)	14.9 (4.5)	0.546
Time with DBS (months)	62.3 (53.4)	44.1 (34.9)	0.187
Levodopa equivalent dosage (mg)	769.9 (533.1)	895.3 (306.6)	0.342
MDS‐UPDRS III in MedOffStimOff (V0)	61.3 (16.7)	61.1 (16.1)	0.978
MDS‐UPDRS III in MedOffStimOn (V0)	36.0 (12.6)	34.2 (11.7)	0.631
MDS‐UPDRS IV (V1)	5.8 (4.1)	9.2 (3.6)	**0.013**
FOG‐AC in MedOnStimOn (V1)	18.0 (7.1)	14.3 (6.3)	0.077
FOG‐Q (V1)	14.2 (4.0)	16.2 (3.4)	0.081
MDS‐UPDRS II 2.13 (*freezing* item) (V1)	3.86 (0.91)	3.73 (0.88)	0.637
CAPSIT‐PD (V1): Time (s)	18.0 (26.0)	8.7 (3.0)	0.105
CAPSIT‐PD (V1): Steps	26.2 (33.1)	14.1 (4.0)	0.095
CAPSIT‐PD (V1): Freezing	1.7 (4.8)	0.1 (0.2)	0.124
Beck's Depression Inventory (V1)	12.5 (9.3)	10.8 (4.0)	0.471
Mini Mental State Examination (V0)	27.8 (2.2)	28.1 (1.9)	0.651
PDQ‐39: Summary score (V1)	35.1 (14.2)	30.6 (10.4)	0.245
Active electrode localization relative to midcommisural point (*x, y, z*)^a^			
Left STN	−12.61 (1.7), −1.05 (1.54), −2.71 (1.45)	−11.57 (1.25), −1.33 (2.33), −3.05 (2.82)	**0.027**, 0.660, 0.632
Right STN	12.09 (1.5), −1.20 (1.58), −3.00 (1.22)	12.33 (1.20), −1.35 (2.64), −3.19 (2.84)	0.554, 0.829, 0.779
Left SNr^b^	−10.48 (1.16), −3.65 (1.43), −6.83 (1.03)	−9.77 (1.15), −4.00 (1.63), −7.32 (1.36)	**0.047**, 0.451, 0.177
Right SNr^b^	10.22 (1.17), −4.00 (1.38), −6.91 (0.95)	10.50 (1.30), −3.82 (1.65), −7.64 (1.29)	0.446, 0.690, **0.038**

Abbreviations: V0 – Screening visit; V1 – baseline visit.

^a^ *x* (medio‐lateral), *y* (anterio‐posterior), *z* (rostro‐caudal) contacts, negative *x* values indicate left‐sided position, negative *y* contacts indicate position posterior to midcommisural point, negative *z* contacts indicate position caudal to the midcommisural point.

^b^ Note that SNr contacts were active only in case of randomization to STN + SNr group after baseline visit V1. However, owing to inclusion criteria and randomization procedure, ventral electrode localization in the ventral STN – SNr border zone to be fulfilled in both treatment arms.

### Patients

We included patients with PD inclusive of genetic forms, disease duration >5 years at the time of study inclusion, age > 18 and <80 years, having received STN‐DBS for ≥6 months, impulse generator Medtronic Activa PC or RC capable of interleaving stimulation, motor improvement of at least 30% with STN stimulation (StimOFF vs. StimON, in MedOFF after overnight withdrawal of dopaminergic medication), Freezing of Gait Assessment Course (FoG‐AC) ≥10 and ≤33, dopaminergic medication constant ≥4 weeks prior to study enrolment, localization of the bilateral active electrodes contact in STN, and localization of the bilateral active electrode contacts in the SNr as expressed by standard coordinates relative to the midcommissural point (left SNr: −7 mm ≤ *x* ≤ −12 mm; −2 mm ≤ *y* ≤ −6 mm; −6 mm ≤ *z* ≤ −10 mm; right SNr: 7 mm ≤ *x* ≤ 12 mm; −2 mm ≤ *y* ≤ −6 mm; −6 mm ≤ *z* ≤ −10 mm). FoG‐AC boundaries were based on (i) clinical reasoning (i.e., we wished to include patients with a sufficient and reproducible amount of FoG (≥10 points) while excluding those who were mostly unable to walk [>33]), and (ii) based on planning data from our previous study, in which we found the strongest effects in patients showing FoG‐AC ≥10 points at baseline.^12^

We excluded patients with MMSE <20 points, acute suicidality or psychosis, pregnancy, in case of comorbidities that interfered with proper conduct of the study or interpretation of the findings (e.g., depression, orthopedic comorbidity, inability to walk outside a freezing episode), presence of paradoxical on‐state freezing,^24^ or participation in another clinical trial within the last 3 months.

### Study Design

The screening visit (V0) included clinical testing in MedOFF to verify the inclusion criteria, but also to ensure optimal individual STN parameters and to allow for reprogramming if appropriate. Then, dopaminergic medication was re‐introduced and the FoG‐AC was assessed in MedON, STN StimON. Thereby, we ensured to study patients in whom FoG proved resistant to STN + medication therapy after having re‐visited and optimized STN parameters. A baseline assessment (Day 1, V1) was performed and patients were randomized in 1:1 ratio to either STN (alone) or STN + SNr using a computer‐generated randomization list. Assessment of the primary endpoint occurred at Day 90 (V6). We performed close interim visits at Day 2 (V2), Day 8 (V3), Day 21 (V4), and Day 42 (V5) to learn about the treatment dynamics of either stimulation paradigm for FoG, that may range from acute^7^ to prolonged effects.^6^, ^12^ Further, tolerance was reported after initially successful programming.^9^

### Physician Roles and Therapy Adjustments

Two DBS expert neurologists were assigned two different roles. The “rating physician” remained blinded to the allocated stimulation program of a patient. After final visit (V6), we asked both the patients and the rating physicians about their assumptions to which of the two treatment arms an individual patient had been randomized based on their personal impression. The answers indicated that blinding was effective, such that both patients’ and physicians’ assumptions were close to chance (patients: randomized to STN group: 12 STN, 11 STN + SNr; STN + SNr group: 12 STN, 10 STN + SNr; physicians: randomized to STN group: 13 STN, 10 STN + SNr; STN + SNr group: 7 STN, 15 STN + SNr).

The second neurologist was the “treating physician,” when performing reprogramming STN parameters in the screening visit (details in Data S1). Further, this physician implemented stimulation parameters following randomization, while keeping the “rating physician” and patient/caregivers blinded. Therefore, the physician was advised to change several times between STN and STN + SNr to mask patients’ insight before maintaining the allocated stimulation program. In case of clinical worsening, adverse events, or safety issues, the treating physician was contacted primarily and decided about any therapy‐adjustments following pre‐specified standards (Data S1).

### Primary Endpoint

The primary endpoint was the FoG‐AC^12^, ^25^ 90 days (V6) after randomization. Clinical details on the FoG‐AC are given as Data S1. It reflects the sum of 12 items ranging in total from 0 to 36 (36 = worst). In addition, we calculated the FoG‐AC change of FoG‐AC(V1) − FoG‐AC (V6); positive values indicated improvement. All assessments took place in MedON, StimON.

### Secondary Endpoints

Secondary endpoints included MDS‐UPDRS II‐IV, CAPSIT‐PD, Berg Balance Scale, Parkinson's disease Questionnaire (PDQ‐39), Freezing of Gait Questionnaire (FOG‐Q), Beck's Depression Inventory, Columbia‐Suicide Severity Rating Scale (C‐SSRS), Clinical global impression (both self‐reported and physician‐reported), MMSE, and falls diary. We asked patients after V6 (before unblinding) whether they wished to continue with their allocated stimulation program.

### Safety Endpoints

The safety population included all patients from the mITT population. Safety variables of patients who withdrew their informed consent during the study were analyzed but not listed with individual data. We defined as safety endpoints: proportion of patients with adverse or serious adverse events (AE/SAE); increase in falls; increase in severity of gait disturbance/FoG over the study course; neuropsychiatric symptoms (i.e., clinically relevant depression, hallucination, psychosis, suicidality); worsening of segmental motor symptoms including akinesia, rigidity, tremor expressed as at least 20% worsening from baseline (arbitrary threshold); increase in dopaminergic on–off fluctuations >20% increase of MDS‐UPDRS IV from baseline.

### Statistical Analysis

We hypothesized that stimulation with “STN+SNr has superior efficacy on FoG‐AC compared to STN alone.” The sample size calculation was based on an effect size of 0.47, a statistical power of 80% and a significance level α = 0.2 (one‐sided t‐test). To detect this improvement of the FoG‐AC (standard deviation of 10.07; mean improvement of 4.67 score points), a sample size of n = 27 patients per therapy group was required (overall n = 54).

### Primary Analysis

We conducted the primary endpoint analysis via ANCOVA with FoG‐AC sum score values at baseline as co‐variable and the FoG‐AC sum score values after 90 days as dependent variable (refer Data S1 for full statistical rationale of primary endpoint analysis). The trial was planned as a non‐confirmatory exploratory study. *P*‐values were not adjusted for multiple testing and used as descriptive measures. The primary analysis was performed for the patients of the modified intention‐to‐treat (mITT) population. The mITT population includes all randomized patients (intention‐to‐treat population (ITT)) without patients who withdraw their informed consent. The ANCOVA examined the null hypothesis that there was no difference in the FoG‐AC sum score after 90 days between the STN and STN + SNr groups: H0: μE = μC versus the alternative hypothesis: the mean FOG‐AC value for the STN + SNr group will be higher than for the STN group: H1: μE > μC. The null hypothesis would be rejected if the probability of error (*P*‐value) of the F‐test fell below the significance level α = 0.200.

As sensitivity analysis, the per‐protocol population was analyzed, which included participants who had no major protocol violation. Major protocol deviations were pre‐defined as experiencing at least one of the following deviations: missing FoG‐AC values after 90 days, given STN‐SNr contacts for group A (STN) and B (STN + SNr) incorrect, coordinates of the active SNr contacts differ >1 mm from the given specifications in the inclusion criteria, malfunction of the stimulator (e.g., impulse generator not working, cable of active contacts broken), clinical factors which did not allow to carry out the study, therapy change for medication or stimulation not according to the protocol.

### Secondary Analyses

For secondary endpoints, the change between V1 and V6 was calculated and the results were compared between the two groups via Mann–Whitney‐U test for quantitative parameters (ordinal data), and via Fisher's Exact test for qualitative parameters (dichotomous data) (all two‐sided significance level α = 0.05). We considered secondary endpoints purely hypothesis‐generating, and therefore did not correct for multiple testing. The exploratory statistical analyses of the secondary endpoints were performed for the mITT population and the per‐protocol population as well.

### Safety Analyses

The safety population includes all patients of the ITT, who received treatment at least once. We analyzed: adverse events (AEs); serious adverse events (SAEs); increase in falls; increase in severity of gait disturbance (FoG); neuropsychiatric symptoms (depression, hallucination/psychosis, suicidality) if judged clinically relevant; worsening of segmental motor symptoms (akinesia, rigidity, tremor >20% worsening from baseline); >20% increase of fluctuations.

### Responder Analyses Based on Minimal Clinically Relevant Change

Further, we conducted responder analyses on the FoG‐AC on both STN and STN + SNr groups with exploratory intent. We defined patients (FoG‐AC, V6 vs V1) as (i) “responders” upon ≥3 points of clinical improvement of the FOG‐AC; (ii) “non‐responders” upon <3 points improvement, unchanged score, or worsening. ≥3 points FoG‐AC improvement reflect minimal clinically relevant change.^25^

### Missing Values

Missing values were not replaced. Statistical testing procedures were run on predefined analysis populations. In case of a missing value the respective value was not analyzed for this specific parameter (“Complete Case Analysis”).

## Results

Here, we report the findings from the mITT population. The sensitivity analysis, using the per‐protocol population, revealed no relevant deviation from the mITT analysis.

### Primary Endpoint

The mITT population showed no relevant difference of the FoG‐AC 90 days after randomization (V6) between “STN” and “STN + SNr” group (unadjusted mean ± SD STN: V1 18.0 ± 7.1, V6 18.1 ± 8.9, STN + SNr: V1 14.3 ± 6.3, V6 14.9 ± 11.1; means adjusted for the baseline FOG‐AC value: STN: V6 17.10, STN + SNr: V6 15.94, adjusted Δ = −1.16, 95%CI [−7.03, 4.71], *P* = 0.692; Hedges g effect size 0.046 95%CI [−0.538, 0.631]) (Fig. 1A).

**Figure 1:**
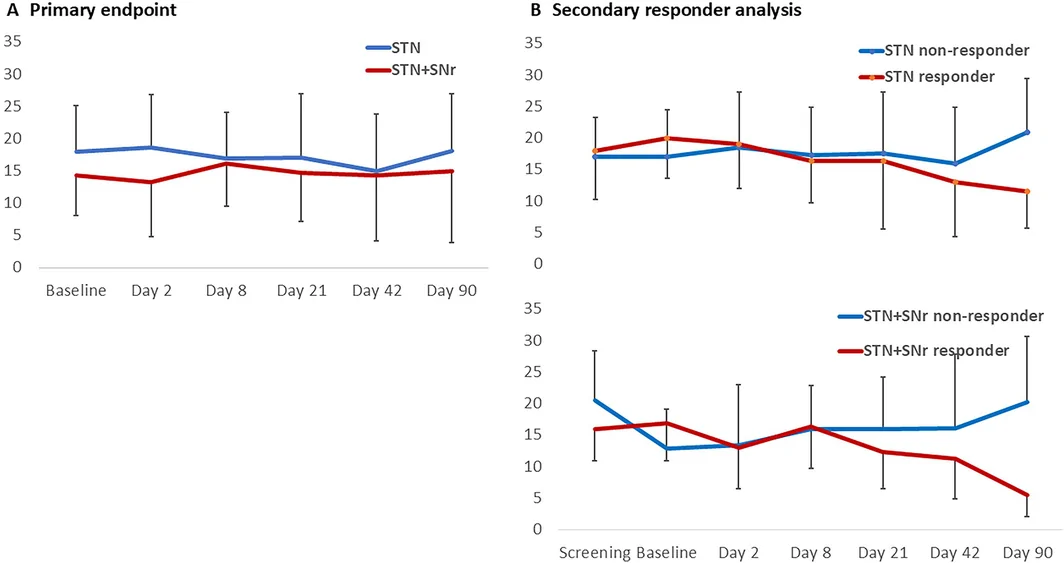
(A) Primary endpoint in the mITT population in terms of the FoG‐AC (mean ± standard deviation). STN group (n = 23) is given in blue, STN + SNr group (n = 22) in red. X‐axis: visits; Y‐axis: Freezing of Gait Assessment Course (FoG‐AC), sum score (range 0–36, worst = 36). (B) Secondary analysis of treatment dynamics of both STN and STN + SNr groups in the mITT population, stratified by non‐responders (blue) and responders (red), respectively; STN responders (n = 7), STN non‐responders (n = 16); STN + SNr responders (n = 8); STN + SNr non‐responders (n = 14). X‐axis: visits. Y‐axis: Freezing of Gait‐Assessment Course (FoG‐AC) (range 0–36) (mean ± standard deviation). Note that all visits were performed in MedON, StimON, and that all screening and baseline visits had been performed in STN (alone) parameters before being randomized to either STN or STN + SNr stimulation following the baseline visit. Unexpectedly, the STN + SNr non‐responders performed substantially better at baseline as compared to screening, keeping in mind that (i) both screening and baseline assessments had taken place with STN (alone) parameters and in medication ON before being randomized to STN + SNr after the baseline assessment. FoG‐AC had been obtained in the screening visit after STN parameters had been optimized, and after dopaminergic medication had been re‐introduced; (ii) medication and stimulation parameters had remained unchanged between screening and baseline visits. The STN + SNr non‐responder group gradually worsened from baseline to Day 90 back to the level of FoG expression that they had experienced during the screening session. Instead, the STN + SNr responders improved gradually.

### Secondary Endpoints

There was no difference in change from V1 to V6 in most secondary endpoints. Number of steps from CAPSIT‐PD showed improvement in STN compared to STN + SNr. Clinical global impression differed between patients and physicians, such that the majority of patients judged their global impression rather worsened compared to baseline, whereas physicians judged it to be equal or slightly improved. Stimulation amplitudes were higher on STN than on SNr contacts based on our reprogramming policy, as expected (refer Table 2 and Data S1 for details). After final follow‐up, more patients in the STN (15/23) than STN + SNr (11/22) group wished not to continue with the stimulation parameters (all descriptive in Table 2).

**TABLE 2:** Summary of the secondary findings

Score	Treatment group	V1 (mean ± SD)	V6 (mean ± SD)	Δ (change ± SD)	*P*‐value*
PDQ‐39 summary score	STN STN + SNr	35.1 ± 14.2 30.6 ± 10.4	36.0 ± 15.7 32.5 ± 11.5	−0.5 ± 12.8 −1.9 ± 7.3	0.735
Freezing of Gait Questionnaire	STN STN + SNr	14.2 ± 4.0 16.2 ± 3.3	14.5 ± 4.7 16.3 ± 2.9	−0.2 ± 4.9 −0.1 ± 1.8	>0.999
MDS‐UPDRS II 2.13 (*freezing* item)	STN STN + SNr	3.86 ± 0.91 3.73 ± 0.88	3.52 ± 1.34 3.91 ± 0.92	−0.29 ± 1.27 0.18 ± 0.91	0.175**
MDS‐UPDRS III	STN STN + SNr	35.2 ± 13.2 36.6 ± 15.2	36.7 ± 12.7 39.5 ± 11.7	−2.2 ± 12.4 −3.1 ± 12.1	0.847
MDS‐UPDRS II	STN STN + SNr	18.0 ± 9.0 18.0 ± 7.2	19.4 ± 9.1 18.4 ± 6.5	−0.7 ± 7.9 −0.4 ± 5.7	0.700
MDS‐UPDRS IV	STN STN + SNr	5.8 ± 4.1 9.2 ± 3.6	5.9 ± 4.2 7.6 ± 2.9	−0.6 ± 4.5 1.6 ± 5.0	0.439
CAPSIT‐PD: “time” (s)	STN STN + SNr	18.0 ± 26.0 8.7 ± 3.0	17.3 ± 30.2 11.1 ± 7.1	0.2 ± 9.9 −2.4 ± 6.7	0.064
CAPSIT‐PD: “number of steps”	STN STN + SNr	26.2 ± 33.1 14.1 ± 4.0	23.1 ± 29.8 16.6 ± 9.1	2.7 ± 7.3 −2.5 ± 7.9	**0.020**
CAPSIT‐PD: “number of freezing episodes”	STN	1.7 ± 4.8	1.0 ± 3.2	0.6 ± 1.7	0.385
	STN + SNr	0.1 ± 0.2	0.5 ± 2.0	−0.4 ± 2.0	
Berg Balance Scale	STN	44.3 ± 8.7	42.5 ± 11.8	0.3 ± 6.9	0.576
	STN + SNr	46.0 ± 6.5	46.4 ± 7.8	−0.4 ± 6.1	
Beck's Depression Inventory	STN	12.5 ± 9.3	15.2 ± 10.4	−2.2 ± 7.5	0.661
	STN + SNr	10.8 ± 4.0	14.8 ± 5.0	−3.2 ± 6.2	
Columbia Suicidality Rating Scale—Suicidal ideation score	STN	1.8 ± 0.4	1.9 ± 0.3	0.1 ± 0.4	0.424
	STN + SNr	1.8 ± 0.4	1.8 ± 0.4	0.0 ± 0.3	
Columbia Suicidality Rating Scale—Suicidal behavior score	STN	2.0 ± 0.2	2.0 ± 0.0	0.0 ± 0.2	0.345
	STN + SNr	2.0 ± 0.0	2.0 ± 0.0	0.0 ± 0.0	
Clinical global impression (change from V1; patients)	STN	‐	3.70 ± 1.41	‐	‐
	STN + SNr	‐	3.68 ± 1.68	‐	‐
Clinical global impression (change from V1; physicians)	STN	‐	4.05 ± 1.20	‐	‐
	STN + SNr	‐	4.45 ± 1.21	‐	‐
Wish to continue with stimulation parameters after V6 (yes/no)	STN	‐	(8 / 15)	‐	‐
	STN + SNr	‐	(11 / 11)	‐	‐
Stimulation amplitude (mA)***	STN (L; R) STN + SNr (STN L; R)	3.2 (1.9, 4.5); 2.9 (1.2, 5.3) 3.7 (1.8, 6.0); 2.9 (1.4, 5.5)	3.0 (1.9, 4.8); 2.9 (1.2, 5.7) 3.6 (1.6, 6.0); 2.8 (1.3, 5.5)	‐ ‐	‐ ‐
	STN + SNr (SNr L; R)****	1.4 (0.7, 2.5); 1.3 (0.7, 2.5)	1.3 (0.5, 2.5); 1.2 (0.5, 2.5)	‐	‐
Levodopa equivalent dosage	STN	769.87 ± 533.06	787.26 ± 527.39	17.39 ± 51.36	0.226**
	STN + SNr	895.30 ± 306.56	977.41 ± 438.46	82.11 ± 238.92	

* Mann–Whitney‐*U*‐Test. Change in clinical global impression (V6 vs. V1) was expressed on a 7‐point Likert scale: 1–3 strong/medium/slight worsening, 4 equal, and 5–7 mild/medium/strong improvement.

** Welch two sample *t*‐test with *P* value and 95%–CI.

*** Stimulation was delivered at 60 μs on STN electrodes (except in two patients) and all SNr contacts. Stimulation amplitudes are given as median (range).

**** Note that STN + SNr group SNr parameters had not been active in baseline, since all patients were treated with STN mono in V1; however, the SNr parameters had been determined in the screening session and were activated in this group following randomization.

### Safety

We observed 27 adverse events in 15 patients of the safety population (N = 47). In the STN group there were 12 AEs, of these 11 (91.7%) were resolved completely, one without follow‐up report. Seven needed no further actions, two were managed by medication adjustment, and three by other actions (no change in medication or stimulation). In the STN + SNr group, there were 15 AEs. Of these, 14 (93.3%) were resolved completely. Four needed no further actions, eight needed adaptation of stimulation parameters, three needed other actions (no change in medication or stimulation).

Eight of the AEs were reported as SAEs, four in STN group and four in STN + SNr. Seven resolved completely, one remained with sequelae (i.e., endocarditis unrelated to study treatment) (AE/SAE summary given as Table 3).

**TABLE 3:** Adverse events

	STN group (N = 24) AE (thereof SAE)	STN + SNr group (N = 23) AE (thereof SAE)
Falls^a^	6 (1)	7 (2)
DBS hardware complications	3 (3)	0
Dyskinesia	0	1 (1)
Bradykinesia	0	1 (0)
Dysarthria	0	1 (0)
Blepharospasm	0	1 (0)
Paresthesia	0	1 (0)
Blurred vision	0	1 (0)
Imbalance	0	1 (0)
Infectious disease	0	1 (1)
Other	3 (0)	0
Sum SAEs	**4**	**4**
Sum any AE	**12**	**15**

^a^ AEs are reported from all patients who received study interventions. We instructed centers to report falls without traumatic injury as AEs only in case frequency and intensity would exceed the pre‐study level of an individual patient.

Based on the falls diary, we used true falls but excluded nearby falls for safety analysis. 14.9% (n = 7) of patients reported at least one fall preceded by FoG (STN n = 4; STN + SNr n = 3), and 8.5% (n = 4) of the patients reported a fall without preceding FoG (STN n = 3; STN + SNr n = 1). Thirteen patients showed worsening of bradykinesia of more than 20% compared to baseline (STN n = 6; STN + SNr n = 7). In 14 patients we identified with a worsening of rigidity of more than 20% (STN n = 8; STN + SNr n = 6). In 15 patients tremor worsened by more than 20% (STN n = 7; STN + SNr n = 8). Twenty‐two patients reported an increase in dopaminergic response fluctuations of more than 20% (12 STN, 10 STN + SNr).

Neuropsychiatric outcomes were stable, such that there were no clinically relevant neuropsychiatric adverse events in either treatment arm. No patient committed suicide. According to the Columbia Suicidality Rating Scale, no patient of the safety population reported suicide plans or suicide attempts. Three patients in STN and four patients in STN + SNr reported a passive “wish to be dead” (CSRS, item 1) since entering the study, without active suicidal thoughts in any patient (CSRS, item 2).

### Responder Analyses Based on Minimal Clinically Relevant Change

Of the entire study cohort, 15 out of 45 patients showed clinically relevant improvement of ≥3 points on the FoG‐AC^25^ at V6 compared to baseline (STN (7/23), STN + SNr (8/22 patients)). In STN + SNr, responders improved compared to non‐responders on the FoG‐AC (14.79 [95% CI: 6.85, 22.72]), and this difference was numerically larger than in STN responders (9.37 [1.96, 16.78]). Interestingly, improvement in both STN and STN + SNr groups evolved gradually over time (Fig. 1B) while the non‐responders showed an inverse pattern.

In STN + SNr, responders had shorter disease duration compared to non‐responders (∆4.5 years, 95%‐CI [0.77, 8.23]), (effect size 1.14, *P* < 0.01). STN responders also showed numerically shorter disease duration compared to non‐responders (∆4.54 95%‐CI [−1.26, 10.33]) and higher FoG‐Q at baseline (∆−3.3 95%‐CI [−6.14, −0.17], *P* = 0.040). Further clinical baseline descriptive of either responder vs. non‐responder group can be depicted from Table 4. Overview on therapy adjustments of responder and non‐responder groups is given as Table S1.

**TABLE 4:** Clinical baseline characteristics of responders and non‐responders of both STN and STN + SNr groups

	STN non‐responder (mean ± SD)	STN responder (mean ± SD)	Δ, [95%‐CI], *P*‐value*	STN + SNr non‐responder (mean ± SD)	STN + SNr responder (mean ± SD)	Δ, [95%‐CI], *P*‐value*
Number of patients (n, %)	16 (69.6)	7 (30.4)		14 (63.6)	8 (36.3)	
Age	68.19 ± 7.84	68.43 ± 7.46	−0.24 [−7.53, 7.05] 0.9458	69.07 ± 6.76	70.75 ± 7.23	−1.68 [−8.09, 4.73] 0.5907
Disease duration (V0) (years)	17.25 ± 6.64	12.71 ± 4.72	4.54 [−1.26, 10.33] 0.1186	16.50 ± 4.20	12.00 ± 3.70	4.50 [0.77, 8.23] **0.0205**
Time with DBS (V0) (months)	70.88 ± 61.74	42.57 ± 15.98	28.30 [−21.53, 78.13] 0.2507	48.86 ± 38.46	35.88 ± 27.96	12.98 [−19.51, 45.47] 0.4144
Levodopa equivalent dosage (LED) (V1) (mg)	873.56 ± 579.61	532.86 ± 328.38	340.71 [−149.68, 831.09] 0.1633	830.07 ± 321.37	1009.44 ± 258.33	−179.37 [−457.46, 98.73] 0.1936
Levodopa equivalent dosage (LED) (V6) (mg)	886.06 ± 570.78	561.43 ± 346.20	325 [−82, 731] 0.111	849.71 ± 326.53	1200.88 ± 537.63	−351 [−817, 115] 0.124
FOG‐AC (V1)	17.06 ± 7.45	20.00 ± 6.35	−2.94 [−9.68, 3.80] 0.3752	12.86 ± 6.24	16.88 ± 5.91	−4.02 [−9.68, 1.65] 0.1545
FOG‐AC (V6)	20.94 ± 8.54	11.57 ± 5.86	9.37 [1.96, 16.78] **0.0157**	20.29 ± 10.35	5.50 ± 3.42	14.79 [6.85, 22.72] **0.0009**
FOG‐Q (V1)	13.13 ± 4.19	16.43 ± 2.70	−3.3 [−6.14, −0.17] **0.040**	16.36 ± 3.59	15.88 ± 3.09	0.48 [−2.6. 3.6] 0.744
FOG‐Q (V6)	13.88 ± 4.57	15.86 ± 5.21	−2.0 [−7.0, 3.1] 0.404	16.29 ± 2.79	16.25 ± 3.41	0.04 [−3.0, 3.1] 0.980
MDS‐UPDRS III in MedOff StimOff (V0)	64.33 ± 16.67 (n missing = 1)	54.43 ± 15.63	9.90 [−5.72, 25.53] 0.2010	61.64 ± 13.33	60.00 ± 21.11	1.64 [−13.59, 16.88] 0.8243
MDS‐UPDRS III in MedOff StimOn (V0)	36.81 ± 9.69	34.86 ± 17.87	1.96 [−9.90, 13.81] 0.7351	35.07 ± 10.87	32.38 ± 14.13	2.70 [−8.50, 13.89] 0.6209
MDS‐UPDRS IV (V1)	6.20 ± 4.35 (n missing = 1)	4.50 ± 3.00 (n missing = 3)	1.70 [−3.22, 6.62] 0.4755	9.18 ± 3.54 (n missing = 3)	9.33 ± 4.03 (n missing = 2)	−0.15 [−4.17, 3.87] 0.9370
Berg Balance Scale (V1)	43.62 ± 9.25 (n missing = 3)	45.83 ± 7.86 (n missing = 1)	−2.22 [−11.45, 7.01] 0.6187	46.71 ± 6.02	44.63 ± 7.41	2.09 [−3.95, 8.13] 0.4792
MMSE (V0)	27.57 ± 2.21 (n missing = 2)	28.14 ± 2.34	−0.57 [−2.75, 1.61] 0.5898	27.93 ± 1.77	28.25 ± 2.12	−0.32 [−2.08, 1.44] 0.7071
Number of medication adjustments from V1‐V6 (number adjustments: number patients)	0: 14	0: 5		0: 11	0: 4	
	1: 1	1: 1		1: 3	1: 2	
	>1: 1	>1: 1		>1: 0	>1: 2	
Number of stimulation adjustments from V1‐V6 (number adjustments: number patients)	0: 10	0: 4		0: 6	0: 4	
	1: 2	1: 3		1: 2	1: 4	
	>1: 4	>1: 0		>1: 6	>1: 0	
L_STN – active contact *x*	−12.69 ± 1.70	−12.43 ± 1.81	−0.26 [−1.89, 1.38] 0.7450	−11.36 ± 1.28	−12.00 ± 1.15 (n missing = 1)	0.64 [−0.56, 1.84] 0.2767
R_STN – active contact *x*	12.06 ± 1.34	12.14 ± 1.95	−0.08 [−1.53, 1.37] 0.9094	12.21 ± 1.31	12.57 ± 0.98 (n missing = 1)	−0.36 [−1.53, 0.82] 0.5332
L_STN – active contact *y*	−1.54 ± 1.45	0 ± 1.26	−1.54 [−2.99, −0.08] **0.0396**	−0.86 ± 2.51	−2.29 ± 1.70 (n missing = 1)	1.43 [−0.78, 3.64] 0.1926
R_STN – active contact *y*	−1.29 ± 0.99	−1 ± 2.61	−0.29 [−1.94, 1.37] 0.7209	−0.54 ± 2.85 (n missing = 1)	−2.86 ± 1.35 (n missing = 1)	2.32 [−0.09, 4.73] **0.0587**
L_STN – active contact *z*	−2.80 ± 1.61	−2.50 ± 1.05	−0.3 [−1.80, 1.20] 0.6805	−2.92 ± 3.30 (n missing = 1)	−3.29 ± 1.80 (n missing = 1)	0.36 [−2.48, 3.21] 0.7920
R_STN – active contact *z*	−3.20 ± 1.26	−2.50 ± 1.05	−0.7 [−1.93, 0.53] 0.2465	−2.93 ± 3.36	−3.71 ± 1.38 (n missing = 1)	0.79 [−2.01, 3.58] 0.5635
L_SNr – active contact *x*	‐	‐	‐	−9.64 ± 1.22	−10.00 ± 1.07	0.36 [−0.72, 1.44] 0.4976
R_SNr – active contact *x*				10.36 ± 1.34	10.75 ± 1.28	−0.39 [−1.61, 0.83] 0.5088
L_SNr – active contact *y*	‐	‐	‐	−3.93 ± 1.77	−4.13 ± 1.46	0.20 [−1.35, 1.74] 0.7935
R_SNr – active contact *y*				−3.29 ± 1.77	−4.75 ± 0.89	1.46 [0.06, 2.87] 0.0422
L_SNr – active contact *z*	‐	‐	‐	−7.36 ± 1.55	−7.25 ± 1.04	−0.11 [−1.39, 1.18] 0.8638
R_SNr – active contact *z*				−7.93 ± 1.38	−7.13 ± 0.99	−0.80 [−1.97, 0.36] 0.1660

*Note*: Patients were classified as dichotomic choice as responders in case of a FoG‐AC improvement ≥3 points reflecting minimal clinically relevant change, all others as non‐responders.

Abbreviations: V0, Screening visit; V1, Baseline visit; V6–90 days follow‐up visit. Electrode coordinates relative to MCP: *x*, mediolateral, *y*, anterioposterior, *z*, rostrocaudal; *unpaired *t*‐test.

In addition, we concatenated all responders and non‐responders across treatment groups to be more sensitive to baseline differences. Overall responders showed shorter disease duration compared to non‐responders (12.33 vs. 16.90; *P* = 0.003). Further, responders showed numerically lower time since DBS implantation, but this did not reach significance (39.00 vs. 60.60 months; statistical trend *P* = 0.061) (Table S2 for summary descriptive).

## Discussion

The aim of this exploratory study was to evaluate the treatment dynamics of STN + SNr and to characterize the clinical characteristics of treatment responders. Patients from pre‐existing DBS cohorts were followed closely over three months. Given the limited sample size and retrospective patient selection, the alpha for the primary endpoint was set to *p* < 0.2, and all statistical results were considered as descriptive rather than confirmatory measures.

On the group‐level, FoG did not differ significantly between STN and STN + SNr, and both treatments were safe. In responder analyses, we identified that about one third of patients in both STN and STN + SNr showed clinically meaningful improvement. The main clinical characteristic was shorter disease duration of the STN + SNr responders. Our findings extend earlier controlled studies and case series,^12^, ^17^, ^19^, ^26^ by showing that, on the group level and in MedON, STN + SNr stimulation cannot be considered more effective than optimized STN for persistent FoG. However, individual patients may benefit from reprogramming both STN and STN + SNr.

### Responder Characteristics

We identified clinically relevant FoG improvement in about one third of patients which is similar to a recent controlled study using STN + SNr showing similar response rate.^20^ Our responder analysis provided several additional insights. Most importantly, responders in STN + SNr and overall responders showed shorter disease duration (STN showed similar numerical difference as statistical trend). However, responders did not differ from non‐responders in terms of STN‐DBS motor response on MDS‐UPDRS III, and measures of cognitive and postural functions. This said, the robust effect of STN‐DBS on motor signs according to the MDS‐UPDRS III widely ruled out the possibility, that STN‐DBS settings may have been suboptimal on motor symptoms. Thus, the presence of FoG in this cohort does not stem from ineffective STN‐DBS. Therefore, our findings prompt the view that both STN and STN + SNr DBS may modulate FoG presumably in disease stages that have not progressed too far, and when presumably the dopaminergic deficit (as expressed by residual dopaminergic fluctuations in both groups) may be the leading pathology. Instead, cognition and gait functions on the CAPSIT‐PD—which may indicate more severe cholinergic pathology^27^ were preserved (refer Table 1). Further, the responder and non‐responder characteristics suggest that the differences in response are not to be explained by dopaminergic fluctuations; firstly, since the severity of fluctuations according to MDS UPDRS IV was similar in responder and non‐responder subgroups of both STN and STN + SNr; secondly, since the treatment dynamics showed a rather monotonous pattern of progressive improvement or worsening (Fig. 1B), respectively.

### Treatment Dynamics

Notably, most of the change in FoG severity in both STN and STN + SNr responder groups occurred with delay from re‐programming, developing constantly between Day 21 and Day 90 (Fig. 1B). While this observation is intriguing and clinically relevant to facilitate long‐term success of any neurostimulation reprogramming for FoG, it also means “not to give up too early” on the potential effect of STN + SNr reprogramming in an individual patient. At the same time, optimal parameter selection upon initial programming seems inherently difficult, since the treating physician cannot necessarily derive immediate clinical readouts on FoG—i.e., (i) whether a patient will or will not respond, and (ii) which stimulation settings of a wide parameter space will provide the highest likelihood to be effective in the following weeks and months to come. Recent studies suggested that kinematic parameters—which were not recorded in this study—could provide an early indication about the individually effective parameters of nigral stimulation.^2^, ^19^, ^28^

Instead, the STN + SNr non‐responder group showed a different pattern of quite profound early improvement of FoG when comparing screening visit and baseline (reflecting before and after STN reprogramming, still prior to randomization). However, this early improvement was gradually lost during the 90 days on STN + SNr and mirrors recent observations of a “vanishing effect” or “tolerance” with “low‐frequency” stimulation of the STN.^9^

### Safety

The safety profile of stimulating SNr in addition to STN was favorable in this study like in previous studies.^10^, ^12^, ^20^ SNr stimulation amplitudes needed to be balanced to avoid acute side effects like paresthesia, oculomotor, or capsular side effects. However, we observed no general worsening of segmental motor symptoms like akinesia or reversal of levodopa effect as was suggested from few anecdotal intraoperative observations. Dyskinesia has been reported only in a minority of patients. While neuropsychiatric side effects were reported exceptionally in other studies,^12^, ^22^, ^29^ we did not observe depressive, hypomanic or hallucination‐psychosis symptoms.

### Methodological Considerations

There are some limitations that could not be controlled for in this study.

#### Disease Characteristics and Phenomenology of FoG

FoG is heterogeneous regarding its clinical characteristics and phenomenology, and this may impact on its amenability to neurostimulation. FoG includes a set of diverse clinical presentations like starting hesitation, shuffling, festination, while passing obstacles, or during turning.^30^ In particular, turning freezes (up to 80%) and freezing while walking straight ahead (up to 20%)^31^ account for the majority of FoG episodes and are well‐represented in the FoG‐AC protocol.^32^ Further, locomotor, cognitive, and emotional characteristics may impact on the circuit processes and amenability of any therapy.^33^, ^34^ Further, we were not able to report the evolution and characteristics of FoG prior to study enrolment including the freezing characteristics before regular surgery and from surgery to study enrolment. Thus, we cannot rule out the possibility that differences in individual disease trajectories may have impacted on our findings in the responder and non‐responder groups.

#### Patient Recruitment

Patients were recruited from pre‐existing cohorts, meaning electrodes were not prospectively implanted but localized retrospectively. Together, with further restrictive selection criteria this did not allow us to obtain the originally intended sample size of 54 patients. With 48 patients enrolled we were close to this number, and it is unlikely that six more patients would have changed the outcome of our primary analysis.

#### Functional Neuroanatomy

Electrode positions based on AC–PC coordinates did not differ between STN + SNr responders and nonresponders. However, detailed comparison of contact locations, tissue activation volumes, or connectomic profiles within a common anatomical space was not feasible because individual MRIs were unavailable under the data‐sharing agreement and intraoperative electrophysiologic data were lacking. Consequently, some caudal contacts may not have been optimally positioned within the SNr, underscoring broader uncertainty regarding its functional organization with diverse projections to brainstem and thalamocortical circuits. Likewise, outcome variability among STN responders may relate to electrode placement within the STN.^35^, ^36^ Nevertheless, evidence is growing in PD that the dorsolateral aspect of the SNr relates to integration of gait, balance, and FoG.^4^, ^12^, ^13^, ^17^, ^19^, ^37^ Intraoperative studies suggested that nigral stimulation resulted in modulation of inhibitory plasticity measures dependent on stimulation frequency and pulse width.^38^, ^39^ Further, nigral neurons may respond differently to motor and cognitive aspects of gait,^40^ and this puts the SNr into an attractive position for tackling FoG which represents a dysexecutive‐motor disorder.

#### Parameter Selection

We optimized STN parameters in a standardized way during the screening visit. However, we could not systematically optimize nigral parameters in sequential clinical work‐up before randomization. Presently, knowledge on optimal nigral reprogramming is limited. Previously, higher or eventually lower frequencies were applied.^17^ In a recent study, we found FoG outcome and “optimal” frequency selection highly variable across patients with most patients showing improvement at SNr frequencies of 71 Hz and 119 Hz, while 30 Hz stimulation was mostly ineffective.^28^ A further unknown is, whether stimulation amplitudes should be provided symmetrically or asymmetrically.^41^ In any case, early surrogates of clinical outcomes like kinematic gait parameters could help optimize the initial SNr programming.^28^

In conclusion, both optimized STN and STN + SNr stimulation were safe and showed similar group‐level outcomes for FoG. Responder analyses indicated that patients with shorter disease duration achieved clinically meaningful improvement. Future effort should focus on (i) stratifying patients by disease characteristics, (ii) refining SNr targeting, and (iii) optimizing parameter selection for FoG.

## Author Roles

(A) Design, (B) Execution, (C) Analysis, (D) Writing, (E) Editing of final version of the manuscript.

D.W.: A, B, C, D, E.

I.C.: B, C, D, E.

M.S.: A, C, D, E.

I.F.: B, C, E.

L.P.: B, E.

P.K.: B, E.

M.P.‐N.: B, E.

W.H.: B, E.

A.A.K.: B, E.

C.J.H.: B, E.

K.B.: B, E.

T.K.: B, E.

M.B.: B, E.

Z.K.: B, E.

S.P.: B, E.

M.H.: B, E.

S.G.: B, E.

T.W.: B, E.

I.C.: B, E.

R.K.: A, B, E.

A.G.: A, B, D, E.

## Collaborators of the STN + SNr study group

We would like to thank further investigators of the STN+SNr study group, including Christian Stallinger (Luxembourg), Frank Hertel (Luxembourg), Daniel Kroneberg (Charite Berlin), Carsten Buhmann, Alessandro Gulberti, Beate Schönewald (Hamburg), Verena Zentsch (Muenster), Verle Visser‐Vandewalle (Cologne).

## Disclosures

**Ethical Compliance Statement:** All study centers received approval of their local Ethic's Committees. All patients participated with written informed consent. Written consent forms were monitored by the Centre for Clinical Studies, University of Tübingen, and archived. Further, the authors of this publication confirm that they have read the Journal's position on issues involved in ethical publication and affirm that this work is consistent with those guidelines.

**Financial Disclosures and Conflicts of Interest:** DW received honoraria as speaker, consultant, and research grants from Abbvie, Abbott, Bial, Boston Scientific, Medtronic, Kyowa Kirin, and Stadapharm. IC reports no conflicts of interest. MS reports no conflicts of interest. IF: consultanties for AbbVie Deutschland and AbbVie Schwitzerland. LP reports no conflicts of interest relevant to this manuscript. PK reports no conflicts of interest. MP‐N received lecture or consulting honoraria from Abbott, Boston Scientific, Medtronic, Abbvie, Bial, Desitin and received study‐related fees from Boston Scientific, Zambon, Abbott. WH received lecture fees and honoraria for serving on advisory boards, travel grants and educational grants from Abbott, Aleva, Boston Scientific, and Medtronic. AAK received honoraria as speaker and consultant from Bial, Boston Scientific, Medtronic and Ipsen. CJH received honoraria and travel support from Alexion Pharma Germany GmbH, Orphalan SA, and Univar Solutions B.V. He received speaker fees from AbbVie, Abbott and Idiogen. KB reports no conflicts of interest. TK reports research funding und speaker fees von Medtronic, Abbott, AbbVie. MB received speaker's honoraria from Medtronic, Boston Scientific, Abbott (formerly St. Jude), FomF, derCampus, GE Medical, UCB, Bial, Apothekerverband Köln e.V., BDN, Ever Pharma, Esteve as well as advisory honoraria for the IQWIG, Medtronic, Esteve, Boston Scientific and Abbvie. ZK was consultant for Bial and Stadapharm, and he received travel compensation from Desitin and Medtronic. SP reports speaker honoraria from Insightec, AbbVie, Medtronic GmbH, and Boston Scientific outside the submitted work and grant/research funding from Parkinson Fonds Deutschland GmbH, and UCB Pharma GmbH. MH reports no conflict of interests. SG reports fundings from patients groups, BMBF, DFG (SPP2177 Radiomics), UM Mainz, Abbott, Boston Scientific, National MS Society, Precisis, Innovationsfond GBA (01NVF22107, INSPIRE – PNRM+); Lectures fees: Abbott, BVDN, IPSEN, Merz Therapeutics, Stadapharm. TW has received honoraria for advisory boards from AbbVie, Archimedes, Phagenesis, Zambon, Bial, Kyowa, STADA, Britannia, Merz, UCB, Mitsubishi Tanabe; honoraria for lectures: Esteve, Britannia, Bial, AbbVie, STADA, UCB, Biogen, Licher, Desitin, Pfizer, Zambon, Teva, Bayer, Ever Pharma, Merz, Neuraxpharm, Esteve; Grants (investigator‐initiated): Licher, AbbVie, Clexio, UCB; Inga Claus received honoraria as speaker, consultant, and research grants from Abbvie, Bial, Desitin, Merz Therapeutics, Stadapharm, and Zambon. RK received speaker's honoraria and/or travel grants from Abbvie, Bial, Desitin, Medtronic and Zambon. AG received research grants from Medtronic, Abbott, Boston Scient. Author disclosures are available in the Supporting Information.

## Supporting information


**Data S1.** Supporting Information.
**Table S1.** Therapy adjustments.
**Table S2.** Comparison of responders and non‐responders concatenated over treatment groups.

**Data S2.** Consort chart.

**Data S3.** ICMJE coi_disclosure_all authors.

## Data Availability

The data that support the findings of this study are available on request from the corresponding author. The data are not publicly available due to privacy or ethical restrictions.

## References

[1] Perez‐Lloret S , Negre‐Pages L , Damier P , et al. Prevalence, determinants, and effect on quality of life of freezing of gait in Parkinson disease. JAMA Neurol 2014;71(7):884–890. 10.1001/jamaneurol.2014.753 24839938

[2] Cebi I , Scholten M , Gharabaghi A , Weiss D . Clinical and kinematic correlates of favorable gait outcomes from subthalamic stimulation. Front Neurol 2020;11:212. 10.3389/fneur.2020.00212 PMC7213078 32431656

[3] Lau B , Meier N , Serra G , et al. Axial symptoms predict mortality in patients with Parkinson disease and subthalamic stimulation. Neurology 2019;92(22):e2559–e2570. 10.1212/WNL.0000000000007562 PMC6556086 31043471

[4] Chastan N , Westby GW , Yelnik J , Bardinet E , do MC , Agid Y , Welter ML . Effects of nigral stimulation on locomotion and postural stability in patients with Parkinson's disease. Brain J Neurol 2009;132(Pt 1):172–184. 10.1093/brain/awn294 19001482

[5] Castrioto A , Lozano AM , Poon YY , Lang AE , Fallis M , Moro E . Ten‐year outcome of subthalamic stimulation in Parkinson disease: a blinded evaluation. Arch Neurol 2011;68(12):1550–1556. 10.1001/archneurol.2011.182 21825213

[6] Barbe MT , Tonder L , Krack P , et al. Deep brain stimulation for freezing of gait in Parkinson's disease with early motor complications. Mov Disord 2020;35(1):82–90. 10.1002/mds.27892 31755599

[7] Moreau C , Defebvre L , Destee A , et al. STN‐DBS frequency effects on freezing of gait in advanced Parkinson disease. Neurology 2008;71(2):80–84. 10.1212/01.wnl.0000303972.16279.46 18420482

[8] Fleury V , Pollak P , Gere J , et al. Subthalamic stimulation may inhibit the beneficial effects of levodopa on akinesia and gait. Movement Dis 2016;31(9):1389–1397. 10.1002/mds.26545 26887333

[9] Ricchi V , Zibetti M , Angrisano S , et al. Transient effects of 80 Hz stimulation on gait in STN DBS treated PD patients: a 15 months follow‐up study. Brain Stimul 2012;5(3):388–392. 10.1016/j.brs.2011.07.001 21824834

[10] Karl JA , Ouyang B , Goetz S , Metman LV . A novel DBS paradigm for axial features in Parkinson's disease: a randomized crossover study. Movement Dis 2020;35(8):1369–1378. 10.1002/mds.28048 32246798

[11] Weiss D , Milosevic L , Gharabaghi A . Deep brain stimulation of the substantia nigra for freezing of gait in Parkinson's disease: is it about stimulation frequency? Parkinsonism Relat Disord 2018;63:229–230. 10.1016/j.parkreldis.2018.12.010 30579819

[12] Weiss D , Walach M , Meisner C , et al. Nigral stimulation for resistant axial motor impairment in Parkinson's disease? A randomized controlled trial. Brain J Neurol 2013;136(Pt 7):2098–2108. 10.1093/brain/awt122 PMC3692032 23757762

[13] Heilbronn M , Scholten M , Schlenstedt C , et al. Anticipatory postural adjustments are modulated by substantia nigra stimulation in people with Parkinson's disease and freezing of gait. Parkinsonism Relat Disord 2019;66:34–39. 10.1016/j.parkreldis.2019.06.023 31272926

[14] Weiss D , Breit S , Wachter T , Plewnia C , Gharabaghi A , Kruger R . Combined stimulation of the substantia nigra pars reticulata and the subthalamic nucleus is effective in hypokinetic gait disturbance in Parkinson's disease. J Neurol 2011;258(6):1183–1185. 10.1007/s00415-011-5906-3 21287187

[15] Brosius SN , Gonzalez CL , Shuresh J , Walker HC . Reversible improvement in severe freezing of gait from Parkinson's disease with unilateral interleaved subthalamic brain stimulation. Parkinsonism Relat Disord 2015;21(12):1469–1470. 10.1016/j.parkreldis.2015.09.047 26482492

[16] Golfre Andreasi N , Rispoli V , Contaldi E , Colucci F , Mongardi L , Cavallo MA , Sensi M . Deep brain stimulation and refractory freezing of gait in Parkinson's disease: improvement with high‐frequency current steering co‐stimulation of subthalamic nucleus and substantia Nigra. Brain Stimul 2020;13(2):280–283. 10.1016/j.brs.2019.10.010 31836466

[17] Valldeoriola F , Munoz E , Rumia J , et al. Simultaneous low‐frequency deep brain stimulation of the substantia nigra pars reticulata and high‐frequency stimulation of the subthalamic nucleus to treat levodopa unresponsive freezing of gait in Parkinson's disease: a pilot study. Parkinsonism Relat Disord 2019;60:153–157. 10.1016/j.parkreldis.2018.09.008 30241951

[18] Tan B , Chen T , Guo P , et al. DBS‐STN&SNr combined stimulation versus DBS‐STN monotarget stimulation for Parkinson's disease freezing: a comparative efficacy study. J Parkinsons Dis 2025;15(7):1264–1274. 10.1177/1877718X251359241 PMC13347519 40760816

[19] Kroneberg D , Al‐Fatly B , Morkos C , Steiner LA , Schneider GH , Kuhn A . Kinematic effects of combined subthalamic and dorsolateral Nigral deep brain stimulation in Parkinson's disease. J Parkinsons Dis 2024;14(2):269–282. 10.3233/JPD-230181 PMC10977420 38363617

[20] Artusi CA , Ledda C , Gallo S , et al. Subthalamic and nigral stimulation for freezing of gait in Parkinson's disease: randomized pilot trial. J Parkinsons Dis 2024;14(8):1602–1613. 10.1177/1877718X241292315 PMC13348324 39957196

[21] Hidding U , Gulberti A , Horn A , et al. Impact of combined subthalamic nucleus and substantia Nigra stimulation on neuropsychiatric symptoms in Parkinson's disease patients. Parkinsons Dis 2017;2017:7306192. 10.1155/2017/7306192 PMC5299199 28246572

[22] Pflug C , Nienstedt JC , Gulberti A , et al. Impact of simultaneous subthalamic and nigral stimulation on dysphagia in Parkinson's disease. Ann Clin Transl Neurol 2020;7(5):628–638. 10.1002/acn3.51027 PMC7261764 32267102

[23] Cebi I , Graf LH , Schutt M , et al. Oral transport, penetration, and aspiration in PD: insights from a RCT on STN + SNr stimulation. Dysphagia 2024;40:811–822. 10.1007/s00455-024-10779-y 39520524

[24] Espay AJ , Fasano A , van Nuenen BF , Payne MM , Snijders AH , Bloem BR . “on” state freezing of gait in Parkinson disease: a paradoxical levodopa‐induced complication. Neurology 2012;78(7):454–457. 10.1212/WNL.0b013e3182477ec0 PMC3466608 22262741

[25] Fietzek UM , Schulz SJ , Ziegler K , Ceballos‐Baumann AO . The minimal clinically relevant change of the FOG score. J Parkinsons Dis 2020;10(1):325–332. 10.3233/JPD-191783 31868684

[26] Wagner JR , Schaper M , Hamel W , et al. Combined subthalamic and Nigral stimulation modulates temporal gait coordination and cortical gait‐network activity in Parkinson's disease. Front Hum Neurosci 2022;16:812954. 10.3389/fnhum.2022.812954 PMC8919031 35295883

[27] Muller ML , Bohnen NI , Kotagal V , et al. Clinical markers for identifying cholinergic deficits in Parkinson's disease. Mov Disord 2015;30(2):269–273. 10.1002/mds.26061 PMC4318774 25393613

[28] Weiss D , Cebi I , Dorrmann L , Löffler M , Klocke P , Schneider M , Gharabaghi A . Nigral stimulation for freezing of gait: kinematic gait parameters inform optimization of stimulation frequency. J Neuroeng Rehabil 2025;22(1):191. 10.1186/s12984-025-01712-x PMC12418677 40926219

[29] Hidding U , Gulberti A , Pflug C , et al. Modulation of specific components of sleep disturbances by simultaneous subthalamic and nigral stimulation in Parkinson's disease. Parkinsonism Relat Disord 2019;62:141–147. 10.1016/j.parkreldis.2018.12.026 30616868

[30] Nutt JG , Bloem BR , Giladi N , Hallett M , Horak FB , Nieuwboer A . Freezing of gait: moving forward on a mysterious clinical phenomenon. Lancet Neurol 2011;10(8):734–744. 10.1016/S1474-4422(11)70143-0 PMC7293393 21777828

[31] Salomon A , Gazit E , Ginis P , et al. A machine learning contest enhances automated freezing of gait detection and reveals time‐of‐day effects. Nat Commun 2024;15(1):4853. 10.1038/s41467-024-49027-0 PMC11156937 38844449

[32] Ziegler K , Schroeteler F , Ceballos‐Baumann AO , Fietzek UM . A new rating instrument to assess festination and freezing gait in parkinsonian patients. Movement Dis 2010;25(8):1012–1018. 10.1002/mds.22993 20310009

[33] Ehgoetz Martens KA , Shine JM , Walton CC , et al. Evidence for subtypes of freezing of gait in Parkinson's disease. Movement Dis 2018;33(7):1174–1178. 10.1002/mds.27417 30153383

[34] Ehgoetz Martens KA , Hall JM , Georgiades MJ , et al. The functional network signature of heterogeneity in freezing of gait. Brain J Neurol 2018;141(4):1145–1160. 10.1093/brain/awy019 29444207

[35] Cherif S , Tempier N , Yeche M , et al. Directional subthalamic deep brain stimulation better improves gait and balance disorders in Parkinson's disease patients: a randomized controlled study. Ann Neurol 2024;97(1):149–162. 10.1002/ana.27099 PMC11683173 39475137

[36] Fan H , Guo Z , Jiang Y , et al. Optimal subthalamic stimulation sites and related networks for freezing of gait in Parkinson's disease. Brain Commun 2023;5(5):fcad238. 10.1093/braincomms/fcad238 PMC10493641 37701817

[37] Scholten M , Klemt J , Heilbronn M , et al. Effects of subthalamic and Nigral stimulation on gait kinematics in Parkinson's disease. Front Neurol 2017;8:543. 10.3389/fneur.2017.00543 PMC5650991 29089922

[38] Milosevic L , Gramer R , Kim TH , et al. Modulation of inhibitory plasticity in basal ganglia output nuclei of patients with Parkinson's disease. Neurobiol Dis 2019;124:46–56. 10.1016/j.nbd.2018.10.020 30391540

[39] Fasano A , Bhowmick SS . Reader response: deep brain stimulation in early‐stage Parkinson disease: five‐year outcomes. Neurology 2021;96(12):590–591. 10.1212/WNL.0000000000011641 33753526

[40] Gulberti A , Wagner JR , Horn MA , et al. Subthalamic and nigral neurons are differentially modulated during parkinsonian gait. Brain J Neurol 2023;146(7):2766–2779. 10.1093/brain/awad006 PMC10316763 36730026

[41] Fasano A , Herzog J , Seifert E , et al. Modulation of gait coordination by subthalamic stimulation improves freezing of gait. Movement Dis 2011;26(5):844–851. 10.1002/mds.23583 21370271

