# Supplementary material for: Disease Duration Differentiates Freezing of Gait Response to Subthalamic and Nigral Stimulation for Parkinson's Disease in an Exploratory Multicenter Controlled Trial
Source: Mov Disord Clin Pract. 2026 Oct 12:10.1002/mdc3.70813. Online ahead of print. doi: 10.1002/mdc3.70813 (PMC13655711; doi:10.1002/mdc3.70813)
Supplement: Supplementary file 2 [file MDC3-9999-0-s001.docx]

**Primary endpoint - Freezing of Gait Assessment Course (FoG-AC)**

The FoG-AC was introduced in detail elsewhere (Ziegler et al. 2010). Briefly, it reflects a short walking parkour performed in clinic. Patients start from a chair performing a transfer from sitting to standing position, initiate walking, turning in place clockwise/counterclockwise, walking through a door, and returning to the chair. The task is repeated three times, i) walking only, ii) walking with second motor task (carrying), and iii) cognitive interference while performing serial subtractions form 100 in steps of seven.

**Reprogramming**

During the screening visit the “treating physician” re-visited the pre-existing STN parameters that a patient had received under clinical routine before entering this study. If appropriate, these parameters were optimized in MedOFF state such that they showed optimal control of segmental motor symptoms in terms of tremor, akinesia, and rigidity. In addition, physicians should adhere to the asymmetry concept when possible, i.e. the STN contralateral to the more bradykinetic side should receive higher electric energy equivalent compared to STN contralateral to the less-affected hemi body side (Fasano et al. 2011). Further, patients should be reprogrammed to constant current mode in case of pre-existing constant-voltage settings. This was intended to assure, that the electric energy delivered would be constant over the entire active study phase. Further, stimulation frequency had to be re-programmed to 125Hz to enable interleaving stimulation in case of treatment allocation to the STN+SNr group. SNr parameters should be determined by bilateral symmetric amplitude increases. Based on empirical evidence, we recommended to increase SNr amplitudes stepwise in a range from 0.7 – 3.0 mA based on clinical efficacy in timed walking based on the subject judgement of the “treating physician” and well below side effect threshold of a SNr contact (Weiss et al. 2013). During the active study phase therapy adjustments were allowed according to the pre-existing treatment adaptation protocol (Supplementary Table 1). First, patients should be kept within their allocated treatment arms. Briefly - in case of FOG worsening - the STN group should undergo asymmetrical increase of the bilateral STN parameters, such that the disease dominant and eventually bradykinetic side could receive increased stimulation. In case there was no relevant akinesia of extremity movement, the better side amplitude should be reduced according to the better side reduction concept (Fasano et al. 2011). Instead, the STN+SNr group should receive an increase of SNr amplitudes primarily. Finally, these suggestions were not mandatory, and we left it to the clinical judgement of each center’s treating physician how to best adapt the individual parameters.

**Statistical analysis of the primary endpoint**

The pre-specified primary analysis in the study protocol was a one-sided t-test of two independent samples (STN+SN, STN alone) of the FoG-AC change 90 days after randomization to baseline. Within the blinded data review the FoG-AC change showed no normal distribution. FoG-AC sum score at baseline and after 90 days demonstrated a moderate correlation (rSpearman = 0.380) and the FoG-AC sum score values at day 90 did not show a skewed distribution. Therefore, a non-parametric method (Mann-Whitney-U test) had to be used for the analysis of the primary endpoint instead of the initially planned one-sided t-test, specified in the study protocol. But according to Vickers a two-sided co-variance analysis (ANCOVA) in this case is not importantly biased compared to the non-parametric Mann-Whitney-U test (Vickers 2005). Effect sizes were calculated as quotients of group differences and pooled baseline standard deviations. Thus, we performed the ANCOVA.

The Hedges g effect size with Feingold correction (Feingold, 2009) and 95% confidence interval was calculated using the unadjusted group difference of the FoG-AC change and the pooled standard deviation of the FoG-AC changes.

**Supplementary Table 1: Therapy adjustments**

|  | **V2-V3** | **V3-V4** | **V4-V5** | **V5-V6** | **Total V2-V6** |
| --- | --- | --- | --- | --- | --- |
| **STN non-responder** |  | | | | |
| Medication adjustments (n) | 0 | 1 | 2 | 1 | 4 |
| Stimulation adjustments (n) | 2 | 3 | 1 | 2 | 8 |
| **STN responder** |  | | | | |
| Medication adjustments (n) | 0 | 1 | 2 | 1 | 4 |
| Stimulation adjustments (n) | 0 | 1 | 2 | 0 | 3 |
| **STN+SNr non-responder** |  | | | | |
| Medication adjustments (n) | 1 | 0 | 0 | 2 | 3 |
| Stimulation adjustments (n) | 2 | 2 | 4 | 5 | 13 |
| **STN+SNr responder** |  | | | | |
| Medication adjustments (n) | 1 | 1 | 0 | 4 | 6 |
| Stimulation adjustments (n) | 3 | 1 | 0 | 0 | 4 |

 Number of therapy adjustments, medication or stimulation in a given time interval. Legend: V2 = Day2, V3 = Day8, V4 = Day21, V5 = Day 42, V6 = Day90

**Supplementary Table 2: Comparison of responders and non-responders concatenated over treatment groups**

We statistically compared the baseline characteristics of responders vs non-responders by concatenating the patients from both treatment groups (STN and STN+SNr pooled together).

| **Baseline characteristics** | **Non-responder**  N = 30*^1^* | **Responder**  N = 15*^1^* | **Difference***^2^* | **95% CI***^2^* | **p-value***^2^* |
| --- | --- | --- | --- | --- | --- |
| **Gender** |  |  |  |  | 0.464*^3^* |
| male | 24 (80%) | 10 (67%) |  |  |  |
| female | 6 (20%) | 5 (33%) |  |  |  |
| **Age** | 68.60 (7.25) | 69.67 (7.17) | -1.1 | -5.7, 3.6 | 0.643 |
| **Disease duration (years)** | 16.90 (5.55) | 12.33 (4.06) | 4.6 | 1.6, 7.5 | **0.003** |
| **Time since DBS implantation (months)** | 60.60 (52.53) | 39.00 (22.63) | 22 | -1.1, 44 | 0.061 |
| **LED** | 853.27 (469.62) | 787.03 (374.36) | 66 | -196, 329 | 0.611 |
| **UPDRS III (MedOff, StimOff)** | 63.14 (14.95) | 57.47 (18.37) | 5.7 | -5.7, 17 | 0.313 |
| **UPDRS III (MedOff, StimOn)** | 35.93 (10.04) | 33.53 (15.44) | 2.4 | -6.8, 12 | 0.592 |
| **UPDRS IV** | 7.46 (4.23) | 7.40 (4.27) | 0.06 | -3.3, 3.4 | 0.970 |
| **FOG-AC** | 15.10 (7.12) | 18.33 (6.11) | -3.2 | -7.4, 0.93 | 0.124 |
| [FOG-AC – Day 90] | 20.63 (9.26) | 8.33 (5.51) | 12 | 7.8, 17 | <0.001 |
| **FOG-Q - Baseline** | 14.69 (4.18) | 16.13 (2.83) | -1.4 | -3.6, 0.71 | 0.183 |
| **CAPSIT-PD – Time, s** | 13.59 (22.39) | 12.84 (9.65) | 0.75 | -9.0, 11 | 0.877 |
| **CAPSIT-PD – steps, n** | 20.43 (28.28) | 19.60 (13.67) | 0.83 | -12, 14 | 0.896 |
| **CAPSIT-PD – FoG episodes, n** | 1.00 (4.12) | 0.60 (1.34) | 0.40 | -1.4, 2.2 | 0.647 |
| **Becks Depression Inventory** | 11.58 (7.98) | 11.86 (5.99) | -0.28 | -4.8, 4.3 | 0.901 |
| **Mini Mental State Examination** | 27.75 (1.97) | 28.20 (2.14) | -0.45 | -1.8, 0.92 | 0.506 |
| **PDQ-39 SI - Baseline** | 32.79 (14.82) | 32.94 (6.40) | -0.16 | -6.6, 6.3 | 0.961 |
| **Stimulation adjustments** |  |  |  |  | 0.705*^3^* |
| 0 | 16 (53%) | 8 (53%) |  |  |  |
| 1 | 8 (27%) | 6 (40%) |  |  |  |
| 2 | 5 (17%) | 1 (6.7%) |  |  |  |
| 3 | 1 (3.3%) | 0 (0%) |  |  |  |
| **Medication adjustments** |  |  |  |  | 0.860*^3^* |
| 0 | 25 (83%) | 9 (60%) |  |  |  |
| 1 | 4 (13%) | 2 (13%) |  |  |  |
| 2 | 1 (3.3%) | 3 (20%) |  |  |  |
| 3 | 0 (0%) | 1 (6.7%) |  |  |  |
| *^1^*n (%); Mean (SD); *^2^*Welch Two Sample t-test or *^3^* Fisher's Exact Test; Abbreviation: CI = Confidence Interval | | | | | |

**References:**

Fasano, A., J. Herzog, E. Seifert, H. Stolze, D. Falk, R. Reese, J. Volkmann, and G. Deuschl. 2011. 'Modulation of gait coordination by subthalamic stimulation improves freezing of gait', *Mov Disord*, 26: 844-51.

Feingold, A. Effect sized for growth-modeling analysis for controlled clinical trials in the same metric as for classical analysis. Psychol Methods 2009;14(1):43-53.

Vickers, A. J. 2005. 'Parametric versus non-parametric statistics in the analysis of randomized trials with non-normally distributed data', *BMC Med Res Methodol*, 5: 35.

Weiss, D., M. Walach, C. Meisner, M. Fritz, M. Scholten, S. Breit, C. Plewnia, B. Bender, A. Gharabaghi, T. Wachter, and R. Kruger. 2013. 'Nigral stimulation for resistant axial motor impairment in Parkinson's disease? A randomized controlled trial', *Brain*, 136: 2098-108.

Ziegler, K., F. Schroeteler, A. O. Ceballos-Baumann, and U. M. Fietzek. 2010. 'A new rating instrument to assess festination and freezing gait in Parkinsonian patients', *Mov Disord*, 25: 1012-8.
