# Supplementary material for: Disease Duration Differentiates Freezing of Gait Response to Subthalamic and Nigral Stimulation for Parkinson's Disease in an Exploratory Multicenter Controlled Trial
Source: Mov Disord Clin Pract. 2026 Oct 12:10.1002/mdc3.70813. Online ahead of print. doi: 10.1002/mdc3.70813 (PMC13655711; doi:10.1002/mdc3.70813)
Supplement: Supplementary file 3 [file MDC3-9999-0-s003.docx]

**Consort chart**

Analyzed (N = 20)

Analyzed (N = 19)

## PP – Analysis

N = 39

**STN+SNr**

Analyzed (N = 23)
♦ Major protocol deviation (N = 3)

Analyzed (N = 22)
♦ Major protocol deviation (N = 3)

## mITT – Analysis

N = 45

## Follow-Up

No follow-up (consent retracted, N = 1)

Randomized 1:1 (ITT)

N = 48

Excluded (N = 5)

♦  Not meeting inclusion criteria (N = 5)

**standard STN**

Screened for eligibility (N = 53)

## Allocation

N = 47

No follow-up (consent retracted, N = 1)

## Enrolment

Allocated to intervention (N = 24)

♦ Received allocated intervention (N = 24)

Allocated to intervention (N = 24)

♦ Received allocated intervention (N = 23)

♦ Did not receive allocated intervention (N = 1; screening failure, erroneously randomized)
